# Interlocking horizontal mattress suture versus Kakiuchi technique in repair of Achilles tendon rupture: a biomechanical study

**DOI:** 10.1007/s10195-017-0455-x

**Published:** 2017-03-15

**Authors:** Matteo Guzzini, Riccardo Maria Lanzetti, Lorenzo Proietti, Daniele Mazza, Mattia Fabbri, Edoardo Monaco, Germano Ferri, Andrea Ferretti

**Affiliations:** Azienda Ospedaliera Sant’Andrea Via di Grottarossa, 1035/1039, 00189 Rome, Italy

**Keywords:** Achilles tendon repair, Interlocking horizontal mattress, Kakiuchi, Biomechanical study

## Abstract

**Background:**

In recent years, the type of surgical treatment for Achilles tendon rupture has been the subject of controversial debate. This biomechanical study evaluates for the first time in literature the ultimate failure load (UFL) of interlocking horizontal mattress (IHM) suture as compared with Kakiuchi suture in Achilles tendon rupture. The hypothesis is that IHM suture can be performed also for Achilles tendon rupture and ensures higher resistance compared with the traditional Kakiuchi suture.

**Materials and methods:**

Twenty fresh bovine Achilles tendons were obtained. Ten preparations were randomly assigned to each of two different groups: group A (10 specimens) sutured by IHM technique, and group B (10 specimens) sutured by Kakiuchi technique. Each construct was mounted and fixed on a tensile testing machine. Static preconditioning of 50 N was applied for 5 min as initial tensioning to stabilize the mechanical properties of the graft, then a load to failure test was performed at crosshead speed of 500 mm/min.

**Results:**

Ten specimens were tested for each group. The mean UFL was 228.6 ± 98.6 N in the IHM suture group and 96.57 ± 80.1 N in the Kakiuchi suture group. Statistical analysis showed a significant difference (*p* < 0.05) with better UFL in the IHM group. In both groups, the failure mode registered in each specimen was suture breakage (rupture of suture thread).

**Conclusions:**

IHM suture achieved better UFL compared with Kakiuchi suture in an animal model of Achilles tendon repair. These results seem to support IHM as a valid option in Achilles tendon rupture.

## Introduction

Achilles tendon injury is a common musculoskeletal disorder that is increasing in frequency, affecting between 5.5 and 9.9 per 100,000 individuals each year in North America [14, 21]. To date, the type of surgical treatment for Achilles tendon rupture has been the subject of controversial debate, and the ideal form of treatment for Achilles tendon rupture remains controversial [23]. For many years, nonoperative treatment was the gold standard, but recent studies have shown that surgical treatment offers advantages over nonsurgical treatment in terms of clinical outcomes and recurrence rate [15]. One of the most important factors in modern surgery for Achilles tendon rupture is the strength of the suture, which is vital to reduce the number of re-ruptures and achieve early rehabilitation. The re-rupture rate is currently about 5 % among those treated with open surgical suture [3, 12, 24]. Early weight-bearing and strengthening exercises are increasingly being used to accelerate recovery [10, 13]. Therefore, strong repair should resist distraction, allowing early rehabilitation and promoting tendon healing [8, 17].

Early mobilization following Achilles tendon repair has been reported to be beneficial in terms of postoperative recovery and improved tendon vascularity [2, 11, 20, 25]. Clinically, patients with early mobilization after Achilles tendon repair have been shown to have shorter rehabilitation time and return-to-sport time [10, 28].

**Fig. 1:**
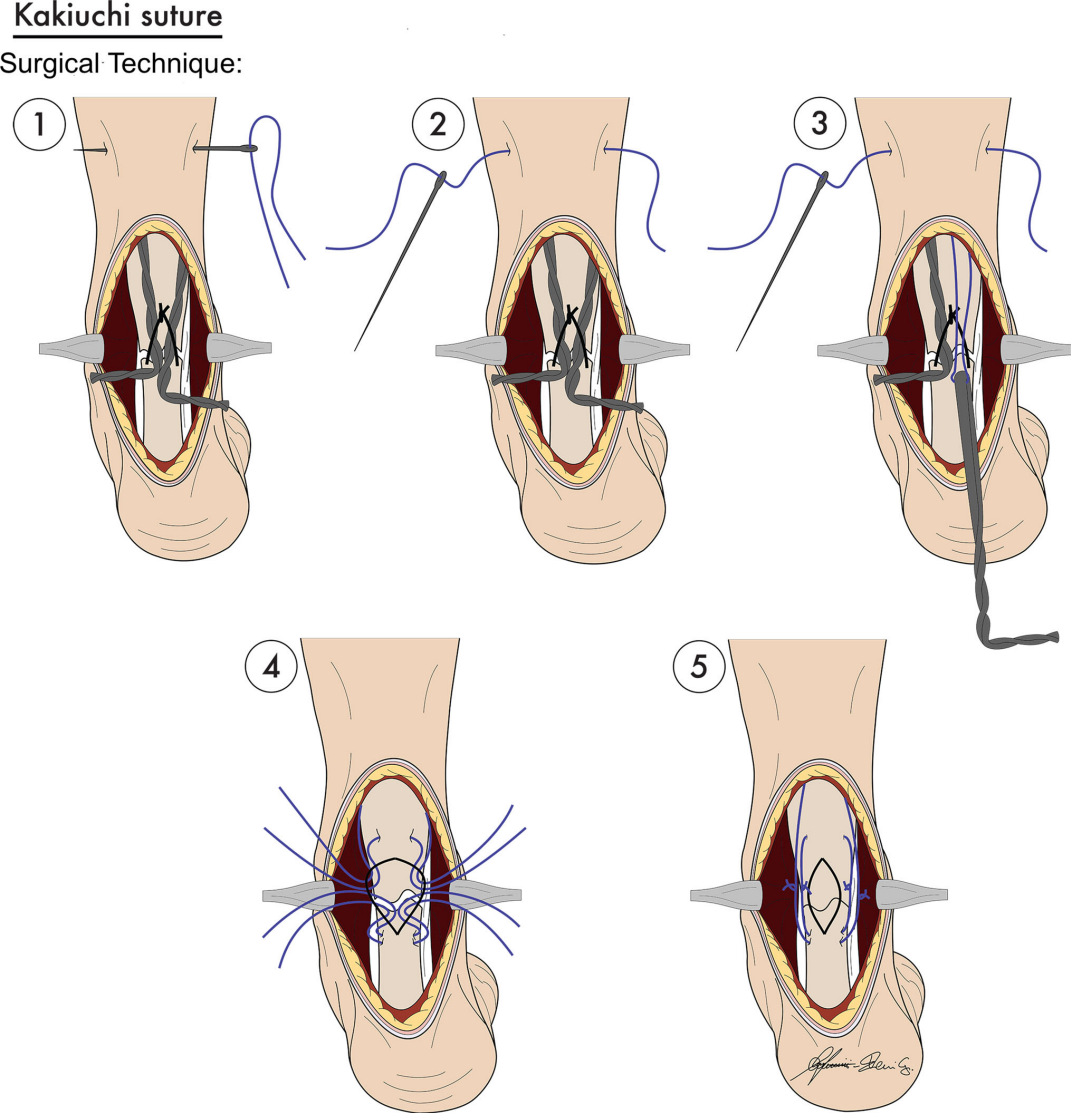
Diagrams of stages of Kakiuchi suture during repair of Achilles tendon rupture

Determining the best suture in terms of strength is one of the most important challenges in this surgery. In this study, it was decided to compare interlocking horizontal mattress (IHM) versus Kakiuchi suture instead of standard techniques such as Kessler, Bunnell or Krackow suture because, nowadays, according to literature [9, 19, 22], Kakiuchi suture is one of the sutures offering greater guarantees in terms of stiffness and tightness in Achilles tendon rupture (Fig. 1).

**Fig. 2:**
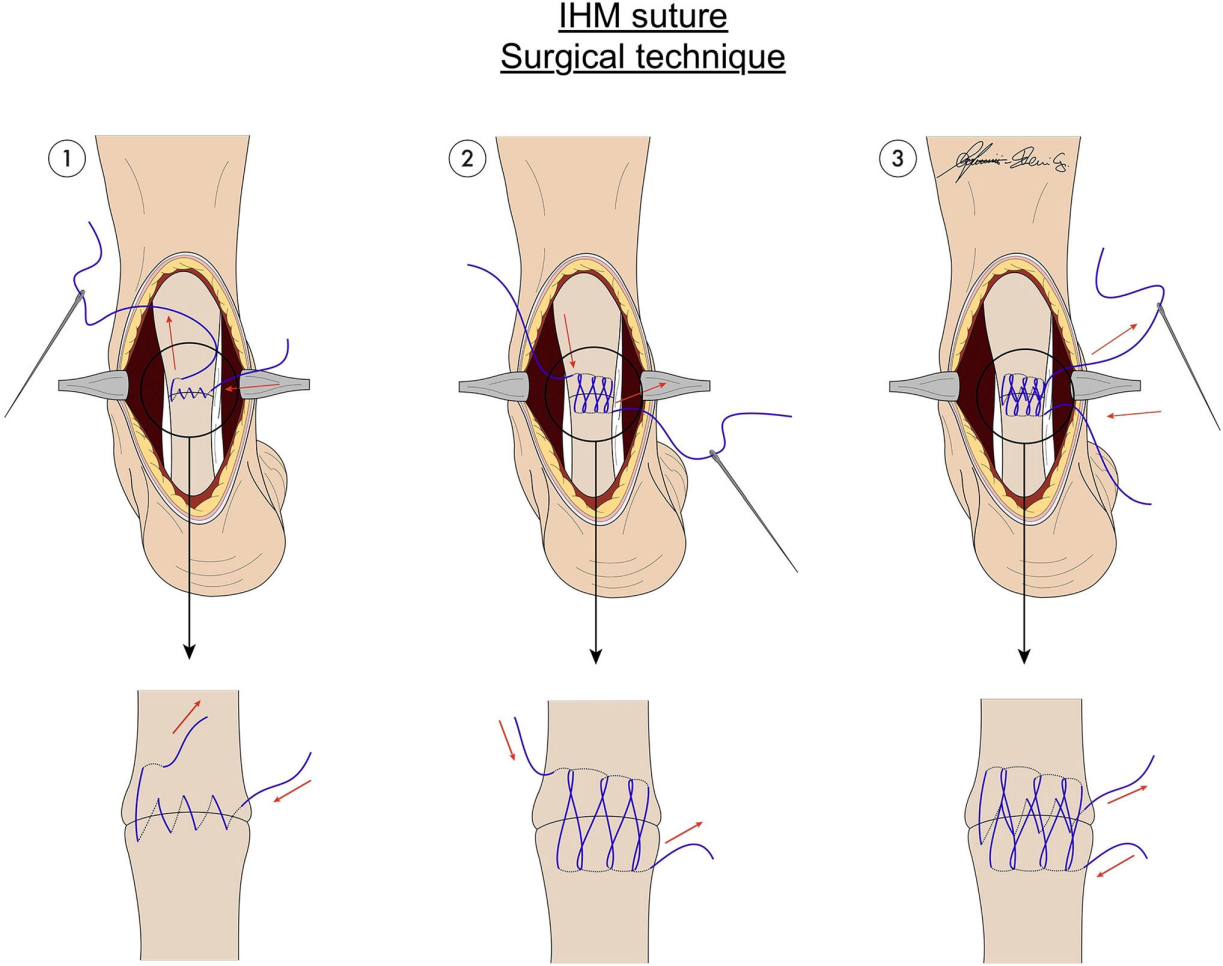
Diagrams of stages of IHM suture during repair of Achilles tendon rupture

Interlocking horizontal mattress suture is the suture giving the best clinical results in literature for hand flexor repair [4]. The IHM suture technique is based on the standard surgical horizontal mattress suture design. With a regular forehand needle insertion, this technique commences on the far side of the repair and proceeds toward the surgeon. This method results in a suture pattern that has its strands running more longitudinally than the oblique patterns of both the cross-stitch and interlocking cross-stitch [5] (Fig. 2).

The aim of the authors is to perform IHM suture in Achilles tendon rupture, a technique never described before in literature.

The aim of this study is to evaluate the biomechanical properties in terms of the ultimate failure load (UFL) of the IHM suture versus the Kakiuchi suture for repair of Achilles tendon rupture. Among the techniques described over the years, there is no literature concerning IHM suture in Achilles tendon rupture. The presented biomechanical study evaluates for the first time in literature the UFL of IHM suture compared with Kakiuchi suture in Achilles tendon rupture. The hypothesis is that IHM could be performed also for Achilles tendon rupture and ensure higher resistance with respect to traditional Kakiuchi suture.

## Materials and methods

Twenty fresh bovine Achilles tendons were obtained from the forefeet of 6-month-old animals. They were kept moist until testing by being wrapped in tissue paper soaked with Ringer’s solution and stored in sealed polyethylene bags. For this study, all applicable international, national, and/or institutional guidelines for the care and use of animals were followed. The same orthopedic surgeon performed tendon harvesting and preparation for testing of each specimen. All tendons were prepared with length of 120 mm, and a lesion was produced in each tendon by using a scalpel to create a clean division in the middle (60 mm). The width and thickness of all tendons were measured and are presented in Table 1. The specimens were not statistically different in terms of width or thickness (*p* < 0.05). Two different suture configurations were tested, using absorbable Ethicon PDS II^®^ (polydioxanone) #2 suture (Fig. 2).

**Table 1:** Width and thickness of constructs

Specimen	Kakiuchi	IHM
	Width, thickness (mm)	Width, thickness (mm)
1	1.1, 0.7	1.5, 0.6
2	1.2, 0.6	1.2, 0.8
3	1.6, 0.8	1.4, 0.6
4	1.5, 0.8	1.2, 0.7
5	1.1, 0.9	1.4, 0.7
6	1.2, 1.1	1.2, 0.9
7	1.4, 0.7	1.1, 0.9
8	1.2, 0.9	1.4, 0.8
9	1.3, 0.7	1.3, 0.7
10	1.3, 0.8	1.5, 0.7
	Mean	Mean
	1.29, 0.79	1.32, 0.74

Ten preparations were randomly assigned to each of two different groups: group A (10 specimens) sutured with IHM technique, and group B (10 specimens) sutured with Kakiuchi technique. Kakiuchi technique is performed by passing transversely through the intact tendon a long straight needle threaded with an absorbable suture, with the same procedure being performed at two different levels on both the proximal and distal stumps of the tendon. Proximally, the suture wire is passed at 0.5 and 1 cm from the cut tendon edge; distally, the suture wire is passed at 0.5 and 1 cm from the rupture site (Fig. 1). In vivo the ankle is held at 30° plantar flexion and the knee flexed to 90° while the sutures are tied. Direct observation confirms that the space between the two stumps is eliminated [9]. The IHM repair technique is performed by running an interlocking horizontal mattress suture, starting at the far end. The suture needle passes underneath the prior crossing suture to lock each throw. When the suture is finished, it is tied at the near end as shown in Fig. 2. In this technique, suture is performed at 0.5 cm from the tendon edge. For IHM, there are six loops for each suture.

**Fig. 3:**
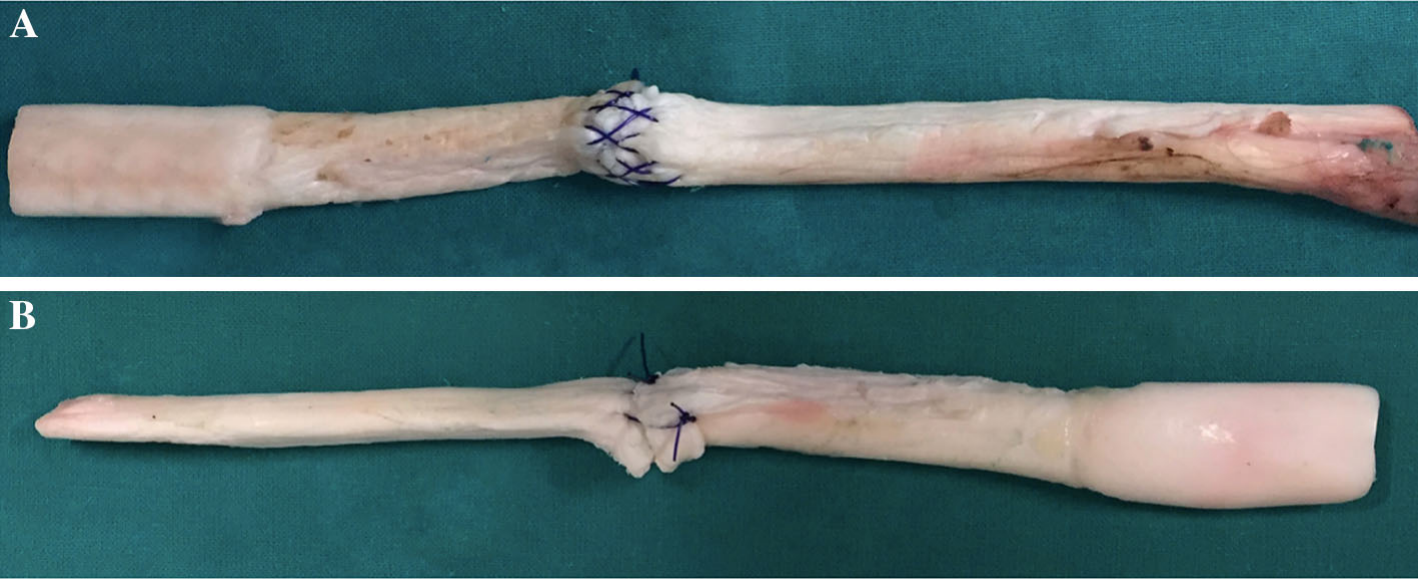
Comparison between IHM suture (**a**) and Kakiuchi suture (**b**) performed on fresh bovine Achilles tendon

**Fig. 4:**
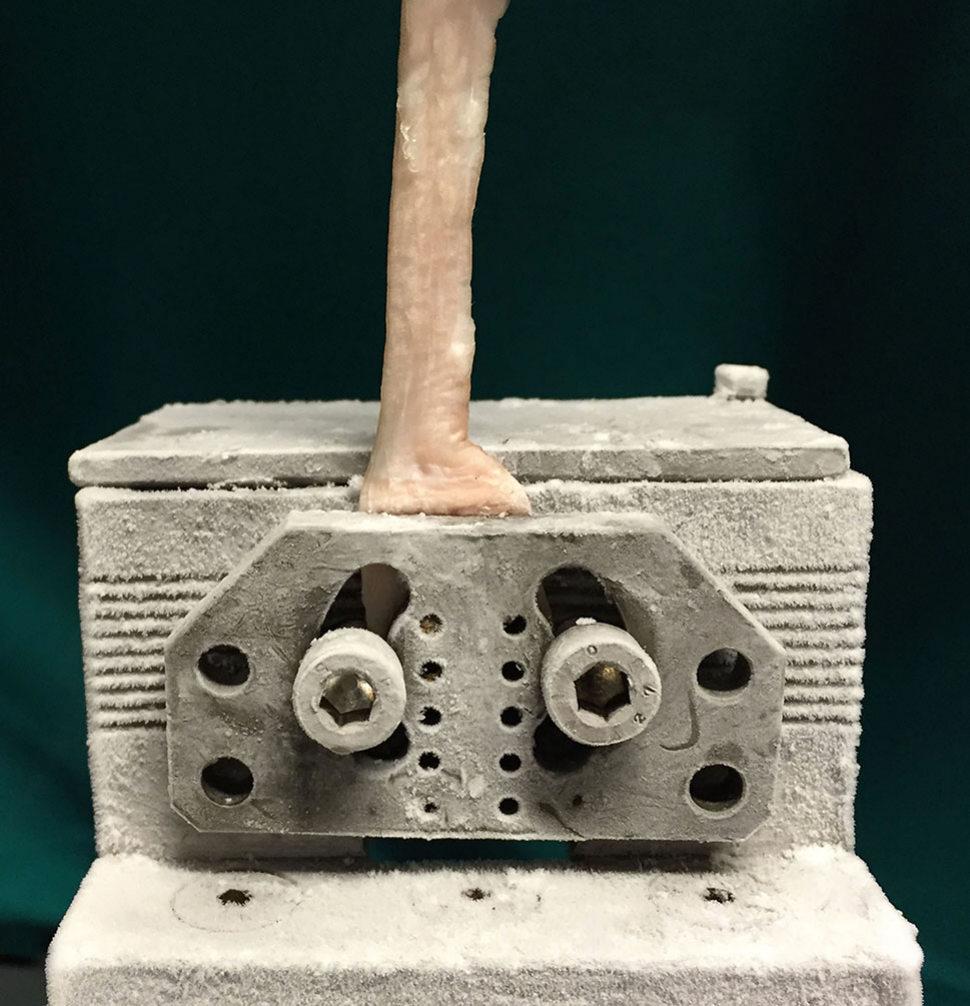
Construct mounted and fixed on a tensile testing machine using two metal clamps connected to the load cell. The specifically designed clamps were frozen to avoid graft slippage from the clamp

**Fig. 5:**
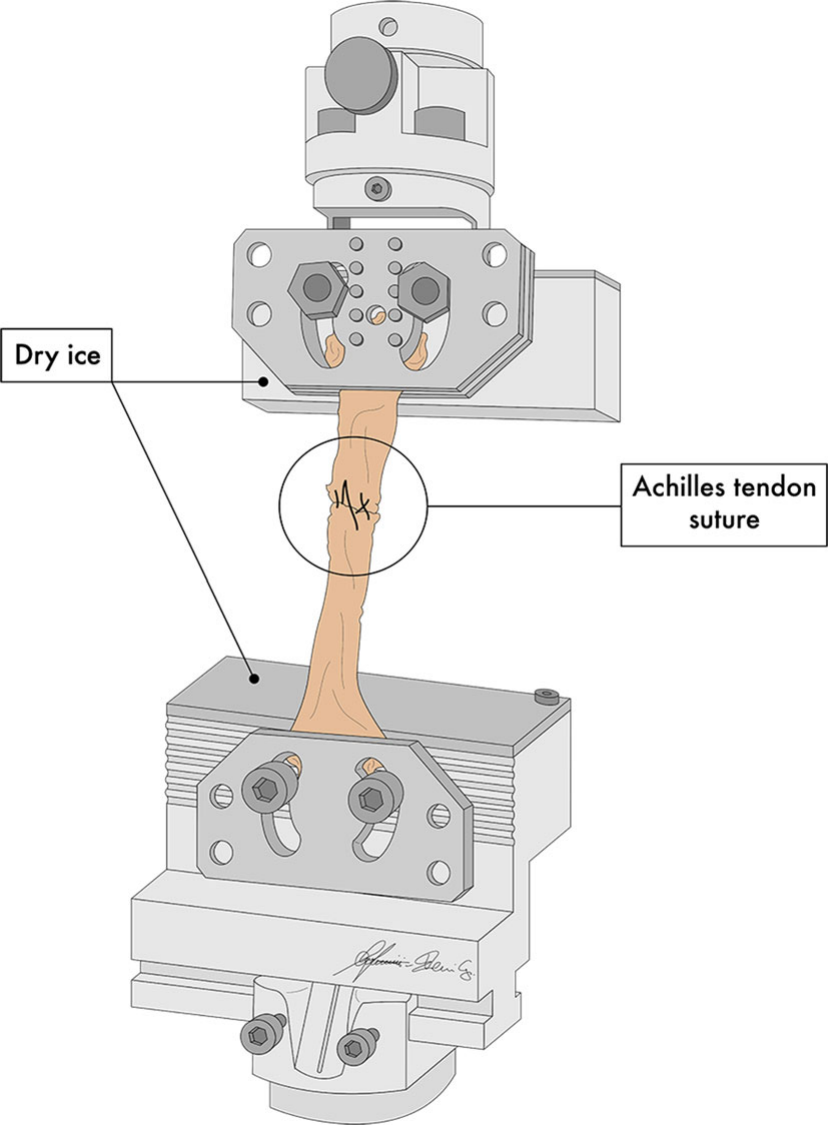
Diagram of construct on tensile testing machine during static preconditioning

**Fig. 6:**
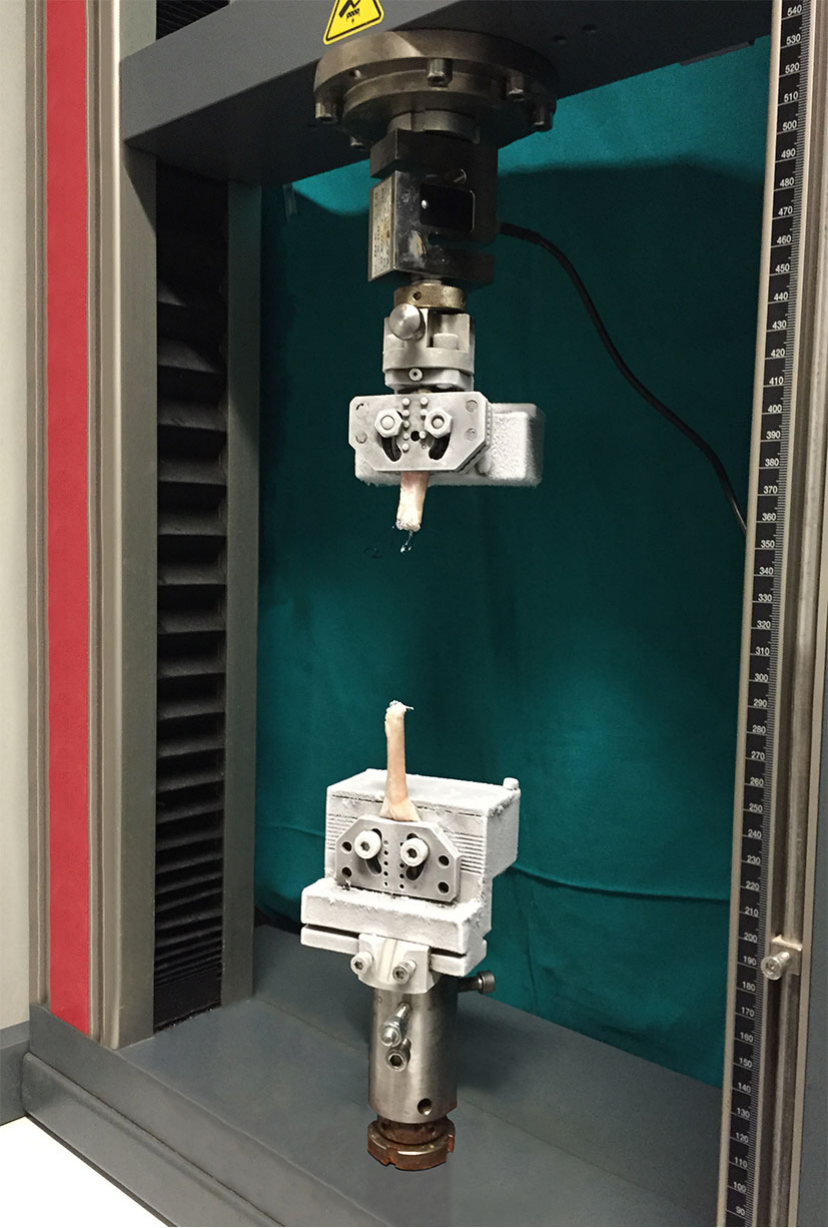
Ultimate failure load (UFL) of construct

Each construct was mounted and fixed on a tensile testing machine (model Z010, Zwick-Ruell, Ulm, Germany) using two metal clamps connected to the load cell. The load was applied parallel to the longitudinal axis of the specimen to obtain the worst load scenario. The specifically designed clamps were frozen to avoid graft slippage from the clamp. Static preconditioning of 50 N was applied for 5 min as initial tensioning to stabilize the mechanical properties of the graft, then a load to failure test was performed at crosshead speed of 500 mm/min. Data regarding the ultimate failure load (UFL) of each specimen were recorded using Textexpert 8.1 software (Zwick-Ruell) and evaluated using a load–displacement curve. We also recorded the failure mode for each construct (Figs. 3, 4, 5, 6).

A total sample size of 18 was considered adequate for overall comparison of the two techniques with respect to construct measurements, assuming an effect size of 0.25, alpha-value of 0.05, and beta-value of 0.20 (study power 80 %).

For all variables, normality of data was ascertained by Kolmogorov–Smirnov test. Differences in construct tensile properties (UFL) between the two groups were compared by Mann–Whitney test. All data were analyzed by a single blinded researcher. The Statistical Package for the Social Sciences (SPSS) version 22 was used for calculations.

## Results

Ten specimens were tested in each group, resulting in a total of 20 specimens. The results are summarized in Table 2. The mean UFL was 228.6 ± 98.6 N in the IHM suture group and 96.5 ± 80.1 N in the Kakiuchi suture group. Statistical analysis showed a significant difference (*p* < 0.05) with better UFL for the IHM group. In both groups, the failure mode registered in each specimen was suture breakage (rupture of suture thread).

**Table 2:** Ultimate failure load (UFL) of the two different suture techniques

Specimen	Kakiuchi	IHM
	UFL (N)	UFL (N)
1	75.89	205.44
2	143.21	201.97
3	70.61	280.40
4	110.12	219.25
5	120.35	196.55
6	63.11	203.40
7	77.02	211.22
8	110.25	300.60
9	113.09	240.94
10	82.02	226.28
	Mean	Mean
	96.57	228.60

## Discussion

The most important finding of this study is that IHM suture exhibited better UFL compared with Kakiuchi suture.

During the early rehabilitation phase, when passive range-of-movement exercises are started, the forces in the Achilles tendon of a healthy limb with the ankle at neutral dorsiflexion range from 70.6 N with the knee in full extension to 17.8 N with the knee flexed to 50° [17] .

Achilles tendon forces at 10° and 20° of ankle dorsiflexion range from 183.2 to 83.4 N and from 401.8 to 215.5 N, respectively, with the knee in full extension and with the knee flexed to 50° [22]. Forces of 190 N are produced when walking in a cam walker with a 1-inch heel raise [1]. When walking, these forces are 2.1 times higher than those exerted by the body weight [6]. The forces on the Achilles tendon during walking and running exceed the strength of all repairs; thus, careful postoperative treatment is imperative [27].

The results presented herein indicate that the IHM technique applied in our study resulted in sufficient primary UFL (228.60 N) to withstand the forces experienced during the early rehabilitation phase, except for passive exercises with 20° of ankle dorsiflexion and knee in full extension (401.8 N).

Another important finding is that we registered a homogeneous mode of failure consisting in suture breakage. No suture slippage across tendon was found, thus we can speculate that both sutures distribute tension across the tendon, avoiding excessive stress that could damage the tendon itself, which has typically undergone degenerative changes in Achilles tendon rupture.

Surgical repair of Achilles tendon rupture is commonly performed, mostly in young active patients, and multiple techniques for treatment of Achilles tendon rupture have been described [7, 26, 28].

The aim of surgical treatment of acute Achilles tendon rupture is to obtain the maximum primary mechanical stability of the sutured tendon, which is crucial to ensure a low rate of postoperative re-rupture and to allow early rehabilitation. For this reason, the type of suture used can affect the clinical outcome and change the postoperative protocol. We tried to determine the load to failure by simulating clinical failure, in which tendon re-rupture occurs in the first 3 months after surgery as a result of increased load. However, this study has several limitations.

First, this was an ex vivo animal study, so we could not reproduce the tendon properties in vivo in humans [16, 18]. We used an apparently healthy tendon, which is different from that usually found in the surgical scenario, where rupture typically appears as a result of degenerative changes involving tissue of the tendon. Moreover, the lesion in the tendon was produced by using a scalpel to create a clean division, in contrast to the frayed ends associated with clinical injury [18]. For all these reasons, only clinical studies might clarify whether IHM suture is truly superior to Kakiuchi suture in the chronic tendinitis scenario. Bovine tendon was tested because it is readily available and has already been used in similar biomechanical studies [18]. Another limitation is that we only recorded data for the UFL, because we aimed only to determine whether IHM could offer better biomechanical performance, considering that graft slippage is more correlated to the characteristics of the tendon itself, which has viscoelastic properties. Finally, the load axis was applied parallel to the graft, which is different from the complex forces that the repaired tendon has to resist during the early rehabilitation phase.

In conclusion, IHM suture exhibited better UFL compared with Kakiuchi suture in an animal model of Achilles tendon repair. These results seem to support IHM as a valid option in Achilles tendon rupture, though the clinical implications of the various repairs were not studied. Many minimally invasive approaches, including for the Kakiuchi technique, have been described in literature for Achilles tendon repair. IHM suture in vivo can also be performed minimally invasively.

More clinical studies are needed to confirm the functional and biomechanical effectiveness of this suture technique.
